# Resting state low-frequency fluctuations in prefrontal cortex reflect degrees of harm avoidance and novelty seeking: an exploratory NIRS study

**DOI:** 10.3389/fnsys.2013.00115

**Published:** 2013-12-17

**Authors:** Takashi Nakao, Tomoya Matsumoto, Daisuke Shimizu, Machiko Morita, Shinpei Yoshimura, Georg Northoff, Shigeru Morinobu, Yasumasa Okamoto, Shigeto Yamawaki

**Affiliations:** ^1^Department of Psychology, Graduate School of Education, Hiroshima UniversityHiroshima, Japan; ^2^Department of Psychiatry and Neurosciences, Institute of Biomedical and Health Sciences, Hiroshima UniversityHiroshima, Japan; ^3^Faculty of Medicine, Hiroshima UniversityHiroshima, Japan; ^4^Department of Psychology, Faculty of Psychology, Otemon Gakuin UniversityOsaka, Japan; ^5^Institute of Mental Health Research, University of OttawaOttawa, ON, Canada; ^6^Department of Neuropsychiatry, Kochi UniversityKochi, Japan

**Keywords:** low-frequency fluctuations, resting state, medial prefrontal cortex (MPFC), personality, reward, aversion, harm avoidance, novelty seeking

## Abstract

Harm avoidance (HA) and novelty seeking (NS) are temperament dimensions defined by Temperament and Character Inventory (TCI), respectively, reflecting a heritable bias for intense response to aversive stimuli or for excitement in response to novel stimuli. High HA is regarded as a risk factor for major depressive disorder and anxiety disorder. In contrast, higher NS is linked to increased risk for substance abuse and pathological gambling disorder. A growing body of evidence suggests that patients with these disorders show abnormality in the power of slow oscillations of resting-state brain activity. It is particularly interesting that previous studies have demonstrated that resting state activities in medial prefrontal cortex (MPFC) are associated with HA or NS scores, although the relation between the power of resting state slow oscillations and these temperament dimensions remains poorly elucidated. This preliminary study investigated the biological bases of these temperament traits by particularly addressing the resting state low-frequency fluctuations in MPFC. Regional hemodynamic changes in channels covering MPFC during 5-min resting states were measured from 22 healthy participants using near-infrared spectroscopy (NIRS). These data were used for correlation analyses. Results show that the power of slow oscillations during resting state around the dorsal part of MPFC is negatively correlated with the HA score. In contrast, NS was positively correlated with the power of resting state slow oscillations around the ventral part of MPFC. These results suggest that the powers of slow oscillation at rest in dorsal or ventral MPFC, respectively, reflect the degrees of HA and NS. This exploratory study therefore uncovers novel neural bases of HA and NS. We discuss a neural mechanism underlying aversion-related and reward-related processing based on results obtained from this study.

## Introduction

Temperament and character are the basic elements of personality that vary among individuals. In contrast to character, which is strongly influenced by experiential factors, temperament is probably more biologically based and stable across a person's life span. Harm avoidance (HA) and novelty seeking (NS) are temperament dimensions defined by the Temperament and Character Inventory (TCI), reflecting a heritable bias for responding intensely to aversive stimuli or for excitement in response to novel stimuli, respectively, (Cloninger, 1987; Cloninger et al., 1993). It is particularly interesting that extreme expression on these temperaments is associated with vulnerability to psychiatric disorders (Richter and Brandstrom, 2009). Increased levels of HA are thought to play a role as a risk factor for development of depression (Joffe et al., 1993; Richter et al., 2000; Farmer et al., 2003; Abrams et al., 2004; Smith et al., 2005; Celikel et al., 2009; Quilty et al., 2010) and anxiety disorders (Jylha and Isometsa, 2006; Mertol and Alkin, 2012). In contrast, a high level of NS is associated with increased risk of exhibiting substance abuse (Cloninger et al., 1988; Gerra et al., 2003) and pathological gambling disorder (Won Kim and Grant, 2001). Therefore, it is important to characterize the biological bases of these temperament traits widely, not only in terms of psychology but of psychiatry.

Neurally, HA and NS are known to be associated with resting state activities in various brain regions including prefrontal cortex (PFC). Positron-emission tomography (PET) reports have described that medial PFC (MPFC) glucose metabolism during resting state is negatively correlated with the HA score (Youn et al., 2002; Hakamata et al., 2006, 2009). Studies measuring cerebral blood flow (Sugiura et al., 2000; O'gorman et al., 2006) also tend to show negative correlation between HA score and activities within frontal regions including MPFC. Functional magnetic resonance imaging (fMRI) studies have demonstrated that functional connectivity between MPFC and amygdala is negatively correlated with the HA score (Li et al., 2012). In contrast, only a few studies have currently addressed the neural characteristics of NS trait from the perspective of resting-state activity. A single photon emission computed tomography (SPECT) study demonstrated that the resting state cerebral blood flow in anterior cingulate and insula are positively correlated with the NS score (Sugiura et al., 2000). Youn and colleagues reported that the NS score is positively associated with the glucose metabolic rate in the right PFC including MPFC (Youn et al., 2002). Taken together, resting state brain activity within MPFC is apparently an important neural basis underlying the temperament traits: HA and NS.

In recent years, interest in the brain's synchronous slow oscillations during a resting state has increased immensely, particularly in the field of psychiatry. Slow oscillations have been observed using measurements of different types, fMRI (Biswal et al., 1995; Fransson, 2006; Chepenik et al., 2010) and electroencephalography (Horovitz et al., 2008; Helps et al., 2010; Broyd et al., 2011; EEG). Although the mechanisms underlying the slow oscillations are not fully understood, slow oscillations of the fMRI blood oxygenation level-dependent (BOLD) signal are known to correlate with local field potentials (LFPs) in a broad frequency range (1–100 Hz) (He et al., 2008; Scholvinck et al., 2010; Pan et al., 2011, 2013; Wang et al., 2012b). Moreover, slow oscillations reportedly modulate higher-frequency activity (Canolty and Knight, 2010; Wang et al., 2012b; Valencia et al., 2013). It is particularly interesting that the slow oscillations have been used to identify the neural characteristics of psychiatric disorders such as major depression disorder (Wang et al., 2012a; Fan et al., 2013; Liu et al., 2013), anxiety disorders (Yin et al., 2011; Hou et al., 2012; Bing et al., 2013), and substance abuse (Jiang et al., 2011). Considering that HA and NS are reported as risk factors for these disorders, it would be interesting to address the question of whether these temperament traits correlate to the slow oscillation activities at rest. However, this question remains to be answered.

This preliminary study was undertaken to characterize the neural bases of temperament dimensions (i.e., HA and NS) by particularly addressing resting state low-frequency fluctuations using near-infrared spectroscopy (NIRS). This non-invasive technique uses near-infrared light to evaluate spatiotemporal characteristics of brain functions near the brain surface. As with fMRI and EEG, NIRS enables the detection of spontaneous slow oscillations in oxygenated hemoglobin (oxy-Hb) (Obrig et al., 2000). Based on earlier studies described above, we specifically focused on the examination of MPFC resting state activity. It is noteworthy that MPFC is characterized by large amplitudes of spontaneous slow oscillations during a resting state (Raichle et al., 2001; Fransson, 2005; Zou et al., 2008). TCI (Cloninger et al., 1993) was used to assess HA and NS temperament traits. We examined whether HA or NS is related with the power of resting-state slow oscillations in the MPFC.

## Method

### Participants

Twenty two healthy volunteer participants (12 males; age range = 21–27 years, mean age = 22.7 years) were recruited from Hiroshima University. All participants were right-handed, with normal or corrected-to-normal vision. All were free of neurological and psychiatric disorders. To control possible confounding factors of brain activity (Duncan and Northoff, 2012), participants who were habitual drinkers or taking medication were not recruited. Participants were not permitted to smoke tobacco from 3 h before the experiment started. Written informed consent was obtained from each participant before the investigation, in line with a protocol approved by the Research Ethics Committee of Hiroshima University. Each participant was paid a small fee for participating.

### Self-report measures

Temperament traits including HA and NS were quantified using the TCI (Cloninger et al., 1993). The TCI is a 240-item questionnaire that assumes a human personality consisting of four temperament and three character dimensions. The temperament dimensions include HA, NS, reward dependence, and persistence. The character dimensions include self-directedness, cooperativeness, and self-transcendence. In this study, the measures of HA and NS were particularly addressed.

### Resting states

After NIRS probe placement, participants were seated on a comfortable chair facing a computer screen in a dark shielded room. During recording, a chin rest was used to help participants maintain the head position. Participants performed counterbalanced resting eyes-closed (EC) and eyes-open (EO) baseline periods of 5 min each. Each participant was instructed to relax and allow the mind to disengage during these periods. During the EO resting state, participants were asked to gaze with fixation at a cross presented at the center of the computer screen, but were allowed to blink normally. Because the EC and EO resting states were thought to reflect baseline brain activity of different types (Marx et al., 2004; Barry et al., 2009; Yan et al., 2009), we included resting states of these two types in the present study. After each type of resting state measurement, participants were asked to fill out a questionnaire that included the question: “Did you fall asleep during the resting state scan?” No participant reported that they had fallen asleep during resting state recordings.

### NIRS data acquisitions

**Figure 1:**
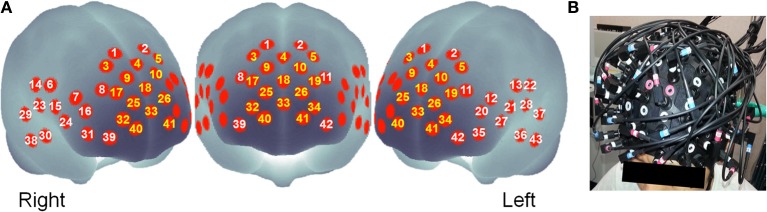
**(A)** Approximate location of the NIRS channel positions in MNI space. The channel number shown in yellow denotes channels of interest for this study, where **(B)** shows the NIRS probe position.

Relative changes in the concentration of oxy-Hb and deoxy-Hb were measured using a multichannel NIRS imaging system (FOIRE-3000; Shimadzu Corp., Kyoto, Japan) with three wavelengths (780, 805, and 830 nm) of infrared light based on Matcher et al. (1995). The data sampling time was 115 ms. The source–detector probes were placed in fronto-temporal regions. The probe set was mounted on a cap for fixation (Figure 1B). The lower frontal probes were positioned along the Fp1–Fp2 line according to the international 10–20 system used for electroencephalography. The distance between pairs of source–detector probes was set at 3 cm. Each measuring area between the pairs of source–detector probes was defined as a channel. It is inferred that the machine, with source–detector spacing of 3 cm, measures points at 2–3 cm depth from the scalp [i.e., measurements are taken from the surface of the cerebral cortex; Hock et al. (1997); Toronov et al. (2001); Okada and Delpy (2003a,b)]. Because the exact optical path length is unknown, the unit used to measure these values is the molar concentration multiplied by length (mM•cm). The 43 measuring points were labeled as ch1–ch43 (see Figure 1A). Of 43 channels, 15 channels in MPFC regions (ch3, ch4, ch5, ch9, ch10, ch17, ch18, ch19, ch25, ch26, ch32, ch33, ch34, ch40, ch41) were used in correlation analyses (see below) for reasons described in the Introduction. Because of a technical problem, data of three channels (ch25, ch28, and ch41) from eight participants failed to record a signal. Unless otherwise indicated, 22 participants' data were used. Three-dimensional locations of the NIRS probe were measured using a Fastrak System (TX-2; Polhemus, USA). Using the MATLAB toolbox NFRI functions (http://www.jichi.ac.jp/brainlab/tools.html), statistical results for each channel were shown for the surface of a standardized brain (Singh et al., 2005).

### NIRS analysis

The NIRS data analysis was conducted using software (MATLAB 8.0; The MathWorks Inc., Natick, MA, USA). Resting state oxy-Hb data were filtered using a low-pass filter of 0.4 Hz. The linear trend caused by drift was removed (Tachtsidis et al., 2004). A Fast Fourier Transform (FFT) was performed on oxy-Hb data EC and EO resting state data. The Welch technique with a Hanning window of 1024 sample points (117.76 s sliding window) and an overlap of 512 points was used. Power spectral density (mM•cm^2^/Hz) was calculated for each channel over the range of 0.02–0.15 Hz. The Welch technique (Welch, 1967) involves sectioning the time-series data into many sub-sections and converting them to a modified estimate of the spectral density before averaging the signals of the sections. Subsequently, the band-limited power in the following two frequency bands was calculated based on previous studies (Obrig et al., 2000; Tachtsidis et al., 2004; Näsi et al., 2011; Pierro et al., 2012): very low-frequency oscillations (VLFO; 0.02–0.04 Hz) and low-frequency oscillations (LFO; 0.04–0.15 Hz). The VLFO and LFO are lower frequency ranges known to be differentiated from other oscillatory phenomena such as eye blinking, heart beat, and respiratory cycles (Obrig et al., 2000; Aminoff, 2012; Pierro et al., 2012; Sassaroli et al., 2012; Li et al., 2012).

### Correlation analysis

To investigate the relations between the temperament traits and resting state activity derived from 15 channels covering MPFC, we performed separate correlation analyses for each combination among temperament traits (HA, NS), different frequency band (VLFO, LFO), and resting states of two types (EC, EO). Before calculating Pearson correlation coefficients, outliers of each datum were excluded from the correlation analysis using an upper limit of the mean ± 3 SD of the participants' data. For cases in which there were outliers for Pearson's correlation analysis, we also calculated Spearman's rank correlation coefficient, which is insensitive to outliers, using all participants' data. In both correlation analyses, Benjamini and Hochberg (BH) false discovery rate (FDR) (Benjamini and Hochberg, 1995) was applied to avoid an increase in false positives for the 15 channels. A bootstrap procedure (Efron and Tibshirani, 1986) with *n* = 1000 resamples was used to establish 95% confidence intervals (*CI*) around the *r* value.

## Results

### Self-report data

The mean scores of HA and NS were, respectively, 51.41 (*SD* = 7.48, range = 35–65) and 48.73 (*SD* = 7.03, range = 36–63). No significant correlation was found between the HA and NS score (*r* = −0.37, *p* = 0.09, *CI* = –0.78–0.13).

### Resting state data

#### Resting state power spectrum density

Table 1 presents the averaged power across all NIRS channels for each resting-state condition (EC and EO) and for each frequency band (VLFO and LFO). The mean VLFO power of the EC resting state was 0.0005 mM•cm^2^/Hz (*SD* = 0.0002). That of the EO resting state was 0.0007 mM•cm^2^/Hz (*SD* = 0.0006). The mean LFO power of the EC resting state was 0.00008 mM•cm^2^/Hz (*SD* = 0.00004). That of the EO resting state was 0.0001 mM•cm^2^/Hz (*SD* = 0.00006). In both frequency bands, the EO resting state showed significantly greater power than the EC resting state did [VLFO, *t*_(21)_ = 2.15, *p* = 0.04; LFO, *t*_(21)_ = 2.98, *p* = 0.007]. These results resemble those reported from earlier studies (Obrig et al., 2000; Tachtsidis et al., 2004; Yan et al., 2009).

**Table 1:** **Summary of averaged power (mM•cm^2^/Hz) across all NIRS channels for each resting state condition (EC and EO) and for each frequency band (VLFO and LFO)**.

		**EC**	**EO**
VLFO	*M*	0.00050	0.00070
	(*SD*)	(0.00020)	(0.00060)
LFO	*M*	0.00008	0.00010
	(*SD*)	(0.00004)	(0.00006)

M, mean; SD, standard deviation; EC, eyes-closed resting state; EO, eyes-open resting state; VLFO, very low-frequency oscillation; LFO, low-frequency oscillation.

These resting state data reported in this manuscript have been published previously (Nakao et al., 2013) and were included as part of a larger data collection. Nakao et al. (2013) reported the results for relations among the power of resting state slow oscillations, early life stress, and frontal activation during decision making tasks. The present manuscript describes a specific examination of the relations between the power of resting state slow oscillations and temperament traits (HA or NS).

#### Correlation between resting state and temperament scores

**Figure 2:**
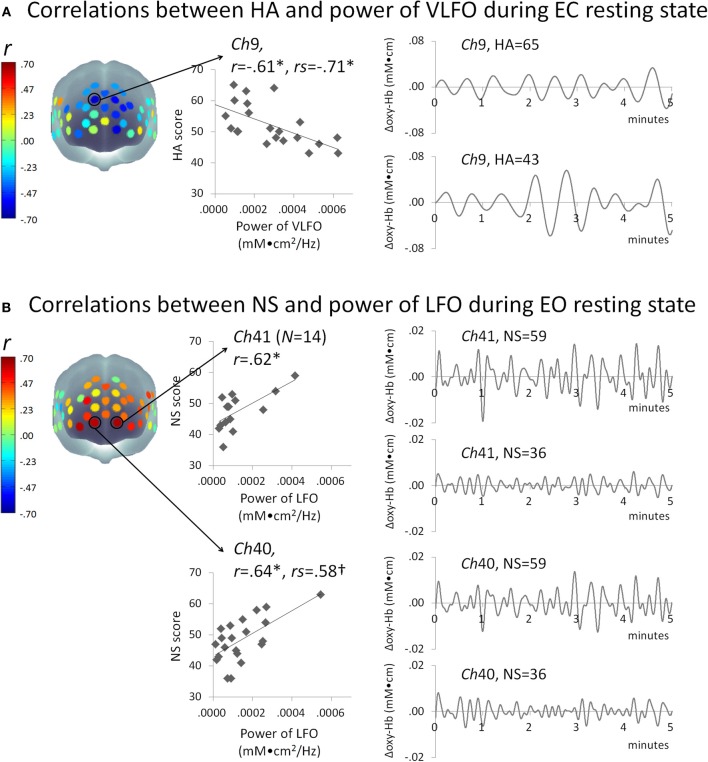
**Schematic figure of correlation results and scatter plots between the powers of resting state slow oscillations (mM•cm^2^/Hz) and (A) HA or (B) NS score.** Waveform plots shown at right are examples of time series data of each frequency range (VLFO, 0.02–0.04 Hz; LFO, 0.04–0.15 Hz) from individuals with high or low temperament trait scores. ^*^FDR adjusted *P* < 0.05; ^†^*FDR* adjusted *P* < 0.07; HA, harm avoidance; NS, novelty seeking; VLFO, very low-frequency oscillation; LFO, low-frequency oscillation; Ch, channel; *r*, Pearson's correlation coefficient; *rs*, Spearman's correlation coefficient.

Figure 2A presents some correlation results between the powers of VLFO during EC resting state and the HA score. The power of VLFO at right dorsal MPFC (ch9, Brodmann area: BA9) was negatively correlated with the HA score (Pearson *r* = −0.61, *FDR* adjusted *p* < 0.05, *CI* = –0.80 to –0.34, *N* = 21; Spearman *r*s = –0.71, *FDR* adjusted *p* < 0.01*CI* = –0.88 to –0.41, *N* = 22). The power of VLFO or LFO during EC or EO resting state in other channels showed no significant correlation with the HA score.

Figure 2B presents the correlation results between the powers of LFO during EO resting state and NS score. The powers of LFO at bilateral ventral MPFC (ch40, BA10; ch41 BA10) were positively correlated with the NS score (ch40, Pearson *r* = 0.64, *FDR* adjusted *p* < 0.05, *CI* = 0.33–0.85, *N* = 21; Spearman *r*s = 0.58, *FDR* adjusted *p* < 0.07 *CI* = 0.19–0.79, *N* = 22; ch41, Pearson *r*= 0.62, *FDR* adjusted *p* < 0.05, *CI* = 0.21–0.90, *N* = 14). The power of VLFO or LFO during EC or EO resting state in other channels showed no significant correlation with NS score.

## Discussion

This study was undertaken to investigate the relations between the power of slow oscillation during resting state and HA or NS. As Figure 2 shows, slow oscillations during resting state at the dorsal MPFC were negatively correlated with the HA score. In contrast, NS was correlated positively with resting-state slow oscillations around the ventral MPFC. These results provide new insights into the neural bases of HA or NS by particularly addressing low-frequency fluctuations.

Previous reports have described that HA is associated with decreased resting state cerebral blood flow (Sugiura et al., 2000; O'gorman et al., 2006) within frontal regions including dorsal MPFC. Although our index of resting state brain activity (i.e., the power of NIRS oxy-Hb slow oscillations) differed from those earlier studies, our results were consistent with those in that HA was found to be associated with the attenuated resting state activity in the dorsal regions of MPFC (Figure 2A). In contrast, our results showed that NS is associated with amplified resting state activation within ventral regions of the MPFC (Figure 2B). These results are consistent with those of previous studies which reported that the NS was associated with increased resting state glucose metabolism in the prefrontal regions including ventral MPFC (Youn et al., 2002). Consequently, these exploratory data provide new evidence that the neural bases of HA or NS can be assessed by low-frequency fluctuations during a resting state measured by NIRS, in addition to other indexes such as the glucose metabolism and cerebral blood flow. It would be interesting to investigate the relations among NIRS low frequency fluctuations and other measurements of brain activity (e.g., the glucose metabolism and cerebral blood flow) in terms of neural bases of temperament traits.

Considering our finding about the relation between HA and the power of resting state slow oscillation, resting state activity in dorsal MPFC might be related to aversion-related processing. Indeed, dorsal MPFC is known as a part of neural network activated by aversive stimuli (Hayes and Northoff, 2011, 2012). The dorsal MPFC is reported to serve an important role in sustaining fear response (Vidal-Gonzalez et al., 2006; Laurent and Westbrook, 2009; Furlong et al., 2010; Robinson et al., 2012). Laurent and Westbrook (2009) demonstrated that inactivation of the rat's paralimbic neurons, which are thought to have similar function with human dorsal MPFC in fear conditioning (Milad et al., 2007, 2009; Robinson et al., 2012), prevents fear response to conditioned aversive stimulus. In addition, Vidal-Gonzalez et al. (2006) demonstrated that microstimulation of that region increased fear response. Robinson et al. (2012) conducted a human fMRI study that showed that the functional connectivity between dorsal MPFC and amygdala was increased during the processing of fearful faces under anxious conditions, and that the amount of coupling was stronger in participants with higher trait anxiety. Based on this evidence, people with high HA personality are expected to show sustained fear response and greater activity in dorsal MPFC under aversive conditions. It would be interesting to examine whether and how the attenuated resting state activity in dorsal MPFC relates to the enhanced aversive-stimulus-induced activity in the same region in high HA people.

Ventral PFC, resting state activity of which correlated positively with NS, is known as a part of the reward-related network (Liu et al., 2011). The activity of ventral PFC is thought to represent the expected value of the outcome which guides reward-based decision making (Hampton and O'Doherty, 2007; O'doherty, 2007; Nakao et al., 2012). Bermpohl et al. (2008) revealed that people with high NS showed enhanced ventral MPFC activity during the expectancy of emotional stimuli. In the relation with resting state brain activity, Li et al. (2013) reported that the resting state functional connectivity in the reward-related network including ventral MPFC was associated with high impulsivity in decision making (i.e., higher preference for an immediate small reward than a larger delayed reward). It is possible that enhanced activity of ventral MPFC at rest observed in people with higher NS scores influences the intensity of the response to rewarding stimuli. Future studies must be undertaken to elucidate how resting state activity in ventral MPFC influences reward-based decision making.

Although we used TCI, which was developed to assess the seven dimensions of the psychobiological model of personality, another line of personality model exists: the five factor model (FFM; Costa and Maccrae, 1992). Neuroticism and extroversion are dimensions of the FFM. These are known to correlate, respectively, with HA and NS (Zuckerman and Cloninger, 1996; De Fruyt et al., 2000; Sher et al., 2000). Like HA, neuroticism is known to be associated with depression and anxiety disorders (Boyce et al., 1991; Rosellini and Brown, 2011). Similarly to NS, a higher extroversion score is associated with alcohol abuse (Flory et al., 2002; Merenäkk et al., 2003). Kunisato et al. (2011) and Wei et al. (2012) examined the relation between resting-state slow oscillation and neuroticism or extroversion using fMRI. They reported that extroversion correlated positively with the amplitude of slow oscillation in the prefrontal regions including ventral MPFC, which are similar to our results for NS. However, they reported no significant correlation between neuroticism and prefrontal regions, which is inconsistent with our results for HA. De Fruyt et al. (2000) reported that 23–51% of the variance of the TCI scales is explainable using the FFM, and concluded that although a substantial overlap exists between the TCI and the FFM, these two cannot be regarded as an equivalent tool to assess individual differences of personality. It would be interesting to examine the differences and similarities between the two personality models in terms of resting state brain activity.

Despite the importance of our data for revealing the neural bases of temperament traits, these findings leave several questions unresolved. First, although NIRS is expected to be useful to assess the bases of HA traits, it was impossible to address the question of how changes of the frontal power of slow oscillation in relation with HA traits are associated with the resting-state activity in the amygdala, where functional connectivity to the MPFC regions was reported previously to correlate to HA (Li et al., 2012; Wang et al., 2013). Additional fMRI studies are expected to be useful to provide further integrative understanding about the neural basis of temperament traits. Second, our data demonstrate that the HA correlated strongly with VLFO power during the EC resting state (Figure 2A), whereas the NS score correlated strongly with LFO power during the EO resting state (Figure 2B). However, although several studies addressed the differences in the frequencies of slow oscillation (Schroeter et al., 2004; Harrison et al., 2008) and the resting state eye conditions (Yang et al., 2007; Qin et al., 2013; Tan et al., 2013), the characteristics in brain function related to these frequencies/conditions remain poorly understood. Further studies investigating the characteristics of VLFO and LFO, and those of EC and EO resting states in the brain function are expected to contribute to the elucidation of the neural bases of temperament traits. Third, we did not record physiological data of eye blink, heat rate, or respiratory cycles because the ranges of slow oscillation can be differentiated from these artifacts (Obrig et al., 2000; Aminoff, 2012; Li et al., 2012; Pierro et al., 2012; Sassaroli et al., 2012). However, recording these artifact data and careful assessment of the pollution on cortical activity data are preferred for future study.

## Conclusion

This study was undertaken to investigate the relations between temperament dimensions (i.e., HA and NS) and the power of slow oscillation in a resting state. We demonstrated a unique relation between them in that HA and NS are oppositely associated, respectively, with the power of slow oscillations in different subregions in the MPFC. These results suggest that the degrees of HA and NS might be predicted by the power of low-frequency fluctuations at rest. Further research on this matter must be conducted using data of more participants. Considering that both slow oscillation activity and temperament traits are involved in the pathophysiology of various psychiatric disorders, the results of this study are expected to be of great interest in the field not only of personality research but also that of psychiatric research. It would therefore be interesting to extend this study to the assessment of patients with such disorders. Beyond elucidating the neural bases of the temperament traits, this line of investigation is expected to contribute to improvement of our understanding of resting-state brain activity.

### Conflict of interest statement

The authors declare that the research was conducted in the absence of any commercial or financial relationships that could be construed as a potential conflict of interest.

## References

[B1] Abrams K. Y. Yune S. K. Kim S. J. Jeon H. J. Han S. J. Hwang J. (2004). Trait and state aspects of harm avoidance and its implication for treatment in major depressive disorder, dysthymic disorder, and depressive personality disorder. Psychiatry Clin. Neurosci. 58, 240–248 10.1111/j.1440-1819.2004.01226.x 15149288

[B2] Aminoff M. J. (2012). Aminoff's Electrodiagnosis in Clinical Neurology, 6th Edn Philadelphia: Elsevier Saunders

[B3] Barry R. J. Clarke A. R. Johnstone S. J. Brown C. R. (2009). EEG differences in children between eyes-closed and eyes-open resting conditions. Clin. Neurophysiol. 120, 1806–1811 10.1016/j.clinph.2009.08.006 19748828

[B4] Benjamini Y. Hochberg Y. (1995). Controlling the false discovery rate: a practical and powerful approach to multiple testing. J. R. Stat. Soc. B 57, 289–300

[B5] Bermpohl F. Pascual-Leone A. Amedi A. Merabet L. B. Fregni F. Wrase J. (2008). Novelty seeking modulates medial prefrontal activity during the anticipation of emotional stimuli. Psychiatry Res. 164, 81–85 10.1016/j.pscychresns.2007.12.019 18703319

[B6] Bing X. Ming-Guo Q. Ye Z. Jing-Na Z. Min L. Han C. (2013). Alterations in the cortical thickness and the amplitude of low-frequency fluctuation in patients with post-traumatic stress disorder. Brain Res. 1490, 225–232 10.1016/j.brainres.2012.10.048 23122880

[B7] Biswal B. Yetkin F. Z. Haughton V. M. Hyde J. S. (1995). Functional connectivity in the motor cortex of resting human brain using echo-planar MRI. Magn. Reson. Med. 34, 537–541 10.1002/mrm.1910340409 8524021

[B8] Boyce P. Parker G. Barnett B. Cooney M. Smith F. (1991). Personality as a vulnerability factor to depression. Br. J. Psychiatry 159, 106–114 10.1192/bjp.159.1.106 1888956

[B9] Broyd S. J. Helps S. K. Sonuga-Barke E. J. S. (2011). Attention-induced deactivations in very low frequency EEG oscillations: differential localisation according to ADHD symptom status. PLoS ONE 6:e17325 10.1371/journal.pone.0017325 21408092 PMC3050980

[B10] Canolty R. T. Knight R. T. (2010). The functional role of cross-frequency coupling. Trends. Cogn. Sci. 14, 506–515 10.1016/j.tics.2010.09.001 20932795 PMC3359652

[B11] Celikel F. C. Kose S. Cumurcu B. E. Erkorkmaz U. Sayar K. Borckardt J. J. (2009). Cloninger's temperament and character dimensions of personality in patients with major depressive disorder. Compr. Psychiatry 50, 556–561 10.1016/j.comppsych.2008.11.012 19840594

[B12] Chepenik L. G. Raffo M. Hampson M. Lacadie C. Wang F. Jones M. M. (2010). Functional connectivity between ventral prefrontal cortex and amygdala at low frequency in the resting state in bipolar disorder. Psychiatry Res. 182, 207–210 10.1016/j.pscychresns.2010.04.002 20493671 PMC2914819

[B13] Cloninger C. (1987). A systematic method for clinical description and classification of personality variants: a proposal. Arch. Gen. Psychiatry 44, 573–588 10.1001/archpsyc.1987.01800180093014 3579504

[B14] Cloninger C. R. Sigvardsson S. Bohman M. (1988). Childhood personality predicts alcohol abuse in young adults. Alcohol. Clin. Exp. Res. 12, 494–505 10.1111/j.1530-0277.1988.tb00232.x 3056070

[B15] Cloninger C. R. Svrakic D. M. Przybeck T. R. (1993). A psychobiological model of temperament and character. Arch. Gen. Psychiatry 50, 975–990 10.1001/archpsyc.1993.01820240059008 8250684

[B16] Costa P. T. Maccrae R. R. (1992). Revised NEO Personality Inventory (NEO PI-R) and NEO Five-Factor Inventory (NEO FFI): Professional Manual. Odessa, FL: Psychological Assessment Resources

[B17] De Fruyt F. Van De Wiele L. Van Heeringen C. (2000). Cloninger's psychobiological model of temperament and character and the five-factor model of personality. Pers. Individ. Dif. 29, 441–452 10.1016/S0191-8869(99)00204-4

[B18] Duncan N. W. Northoff G. (2012). Overview of potential procedural and participant-related confounds for neuroimaging of the resting state. J. Psychiatry Neurosci. 37:120059 10.1503/jpn.120059 22964258 PMC3581596

[B19] Efron B. Tibshirani R. (1986). Bootstrap methods for standard errors, confidence intervals, and other measures of statistical accuracy. Stat. Sci. 1, 54–75 10.1214/ss/1177013815

[B20] Fan T. Wu X. Yao L. Dong J. (2013). Abnormal baseline brain activity in suicidal and non-suicidal patients with major depressive disorder. Neurosci. Lett. 534, 35–40 10.1016/j.neulet.2012.11.032 23201633

[B21] Farmer A. Mahmood A. Redman K. Harris T. Sadler S. McGuffin P. (2003). A sib-pair study of the temperament and character inventory scales in major depression. Arch. Gen. Psychiatry 60, 490–496 10.1001/archpsyc.60.5.490 12742870

[B22] Flory K. Lynam D. Milich R. Leukefeld C. Clayton R. (2002). The relations among personality, symptoms of alcohol and marijuana abuse, and symptoms of comorbid psychopathology: results from a community sample. Exp. Clin. Psychopharmacol. 10, 425–434 10.1037/1064-1297.10.4.425 12498340

[B23] Fransson P. (2005). Spontaneous low-frequency BOLD signal fluctuations: an fMRI investigation of the resting-state default mode of brain function hypothesis. Hum. Brain Mapp. 26, 15–29 10.1002/hbm.20113 15852468 PMC6871700

[B24] Fransson P. (2006). How default is the default mode of brain function? Further evidence from intrinsic BOLD signal fluctuations. Neuropsychologia 44, 2836–2845 10.1016/j.neuropsychologia.2006.06.017 16879844

[B25] Furlong T. M. Cole S. Hamlin A. S. McNally G. P. (2010). The role of prefrontal cortex in predictive fear learning. Behav. Neurosci. 124, 574–586 10.1037/a0020739 20939658

[B26] Gerra G. Bassignana S. Zaimovic A. Moi G. Bussandri M. Caccavari R. (2003). Hypothalamic–pituitary–adrenal axis responses to stress in subjects with 3,4-methylenedioxy-methamphetamine (‘ecstasy’) use history: correlation with dopamine receptor sensitivity. Psychiatry Res. 120, 115–124 10.1016/S0165-1781(03)00175-6 14527643

[B27] Hakamata Y. Iwase M. Iwata H. Kobayashi T. Tamaki T. Nishio M. (2006). Regional brain cerebral glucose metabolism and temperament: a positron emission tomography study. Neurosci. Lett. 396, 33–37 10.1016/j.neulet.2005.11.017 16356648

[B28] Hakamata Y. Iwase M. Iwata H. Kobayashi T. Tamaki T. Nishio M. (2009). Gender difference in relationship between anxiety-related personality traits and cerebral brain glucose metabolism. Psychiatry Res. 173, 206–211 10.1016/j.pscychresns.2008.10.002 19682867

[B29] Hampton A. N. O'Doherty J. P. (2007). Decoding the neural substrates of reward-related decision making with functional MRI. Proc. Natl. Acad. Sci. U.S.A. 104, 1377–1382 10.1073/pnas.0606297104 17227855 PMC1783089

[B30] Harrison B. J. Pujol J. Ortiz H. Fornito A. Pantelis C. Yücel M. (2008). Modulation of brain resting-state networks by sad mood induction. PLoS ONE 3:e1794 10.1371/journal.pone.0001794 18350136 PMC2263138

[B31] Hayes D. J. Northoff G. (2011). Identifying a network of brain regions involved in aversion-related processing: a cross-species translational investigation. Front. Integr. Neurosci. 5:49 10.3389/fnint.2011.00049 22102836 PMC3215229

[B32] Hayes D. J. Northoff G. (2012). Common brain activations for painful and non-painful aversive stimuli. BMC Neurosci. 13:60 10.1186/1471-2202-13-60 22676259 PMC3464596

[B33] He B. J. Snyder A. Z. Zempel J. M. Smyth M. D. Raichle M. E. (2008). Electrophysiological correlates of the brain's intrinsic large-scale functional architecture. Proc. Natl. Acad. Sci. U.S.A. 105, 16039–16044 10.1073/pnas.0807010105 18843113 PMC2564983

[B34] Helps S. K. Broyd S. J. James C. J. Karl A. Chen W. Sonuga-Barke E. J. (2010). Altered spontaneous low frequency brain activity in attention deficit/hyperactivity disorder. Brain Res. 1322, 134–143 10.1016/j.brainres.2010.01.057 20117101

[B35] Hock C. Villringer K. Müller-Spahn F. Wenzel R. Heekeren H. Schuh-Hofer S. (1997). Decrease in parietal cerebral hemoglobin oxygenation during performance of a verbal fluency task in patients with Alzheimer's disease monitored using near-infrared spectroscopy (NIRS)—correlation with simultaneous rCBF-PET measurements. Brain Res. 755, 293–303 10.1016/S0006-8993(97)00122-4 9175896

[B36] Horovitz S. G. Fukunaga M. De Zwart J. A. Van Gelderen P. Fulton S. C. Balkin T. J. (2008). Low frequency BOLD fluctuations during resting wakefulness and light sleep: a simultaneous EEG-fMRI study. Hum. Brain Mapp. 29, 671–682 10.1002/hbm.20428 17598166 PMC6871022

[B37] Hou J. Wu W. Lin Y. Wang J. Zhou D. Guo J. (2012). Localization of cerebral functional deficits in patients with obsessive-compulsive disorder: a resting-state fMRI study. J. Affect. Disord. 138, 313–321 10.1016/j.jad.2012.01.022 22331021

[B38] Jiang G. H. Qiu Y. W. Zhang X. L. Han L. J. Lv X. F. Li L. M. (2011). Amplitude low-frequency oscillation abnormalities in the heroin users: a resting state fMRI study. Neuroimage 57, 149–154 10.1016/j.neuroimage.2011.04.004 21515385

[B39] Joffe R. T. Bagby R. M. Levitt A. J. Regan J. J. Parker J. D. (1993). The tridimensional personality questionnaire in major depression. Am. J. Psychiatry 150, 959–960 8494077 10.1176/ajp.150.6.959

[B40] Jylha P. Isometsa E. (2006). Temperament, character and symptoms of anxiety and depression in the general population. Eur. Psychiatry 21, 389–395 10.1016/j.eurpsy.2005.09.003 16360306

[B41] Kunisato Y. Okamoto Y. Okada G. Aoyama S. Nishiyama Y. Onoda K. (2011). Personality traits and the amplitude of spontaneous low-frequency oscillations during resting state. Neurosci. Lett. 492, 109–113 10.1016/j.neulet.2011.01.067 21291958

[B42] Laurent V. Westbrook R. F. (2009). Inactivation of the infralimbic but not the prelimbic cortex impairs consolidation and retrieval of fear extinction. Learn. Mem. 16, 520–529 10.1101/lm.1474609 19706835

[B43] Li N. Ma N. Liu Y. He X. S. Sun D. L. Fu X. M. (2013). Resting-state functional connectivity predicts impulsivity in economic decision-making. J. Neurosci. 33, 4886–4895 10.1523/JNEUROSCI.1342-12.2013 23486959 PMC6618998

[B44] Li Y. Qin W. Jiang T. Zhang Y. Yu C. (2012). Sex-dependent correlations between the personality dimension of harm avoidance and the resting-state functional connectivity of amygdala subregions. PLoS ONE 7:e35925 10.1371/journal.pone.0035925 22558274 PMC3338761

[B45] Liu C.-H. Ma X. Wu X. Fan T.-T. Zhang Y. Zhou F.-C. (2013). Resting-state brain activity in major depressive disorder patients and their siblings. J. Affect. Disord. 149, 299–306 10.1016/j.jad.2013.02.002 23474094

[B46] Liu X. Hairston J. Schrier M. Fan J. (2011). Common and distinct networks underlying reward valence and processing stages: a meta-analysis of functional neuroimaging studies. Neurosci. Biobehav. Rev. 35, 1219–1236 10.1016/j.neubiorev.2010.12.012 21185861 PMC3395003

[B47] Marx E. Deutschlander A. Stephan T. Dieterich M. Wiesmann M. Brandt T. (2004). Eyes open and eyes closed as rest conditions: impact on brain activation patterns. Neuroimage 21, 1818–1824 10.1016/j.neuroimage.2003.12.026 15050602

[B48] Matcher S. J. Elwell C. E. Cooper C. E. Cope M. Delpy D. T. (1995). Performance comparison of several published tissue near-infrared spectroscopy algorithms. Anal. Biochem. 227, 54–68 10.1006/abio.1995.1252 7668392

[B49] Merenäkk L. Harro M. Kiive E. Laidra K. Eensoo D. Allik J. (2003). Association between substance use, personality traits, and platelet MAO activity in preadolescents and adolescents. Addict. Behav. 28, 1507–1514 10.1016/S0306-4603(02)00270-8 14512074

[B50] Mertol S. Alkin T. (2012). Temperament and character dimensions of patients with adult separation anxiety disorder. J. Affect. Disord. 139, 199–203 10.1016/j.jad.2012.02.034 22440427

[B51] Milad M. R. Pitman R. K. Ellis C. B. Gold A. L. Shin L. M. Lasko N. B. (2009). Neurobiological basis of failure to recall extinction memory in posttraumatic stress disorder. Biol. Psychiatry 66, 1075–1082 10.1016/j.biopsych.2009.06.026 19748076 PMC2787650

[B52] Milad M. R. Quirk G. J. Pitman R. K. Orr S. P. Fischl B. Rauch S. L. (2007). A role for the human dorsal anterior cingulate cortex in fear expression. Biol. Psychiatry 62, 1191–1194 10.1016/j.biopsych.2007.04.032 17707349

[B53] Nakao T. Matsumoto T. Morita M. Shimizu D. Yoshimura S. Northoff G. (2013). The degree of early life stress predicts decreased medial prefrontal activations and the shift from internally to externally guided decision making: an exploratory NIRS study during resting state and self-oriented task. Front. Hum. Neurosci. 7:339 10.3389/fnhum.2013.00339 23840186 PMC3699719

[B54] Nakao T. Ohira H. Northoff G. (2012). Distinction between externally vs. internally guided decision-making: operational differences, meta-analytical comparisons and their theoretical implications. Front. Neurosci. 6:31 10.3389/fnins.2012.00031 22403525 PMC3293150

[B55] Näsi T. Virtanen J. Noponen T. Toppila J. Salmi T. Ilmoniemi R. J. (2011). Spontaneous hemodynamic oscillations during human sleep and sleep stage transitions characterized with near-infrared spectroscopy. PLoS ONE 6:e25415 10.1371/journal.pone.0025415 22043284 PMC3197192

[B56] Obrig H. Neufang M. Wenzel R. Kohl M. Steinbrink J. Einhaupl K. (2000). Spontaneous low frequency oscillations of cerebral hemodynamics and metabolism in human adults. Neuroimage 12, 623–639 10.1006/nimg.2000.0657 11112395

[B57] O'doherty J. P. (2007). Lights, camembert, action! The role of human orbitofrontal cortex in encoding stimuli, rewards, and choices. Ann. N.Y. Acad. Sci. 1121, 254–272 10.1196/annals.1401.036 17872386

[B58] O'gorman R. L. Kumari V. Williams S. C. Zelaya F. O. Connor S. E. Alsop D. C. (2006). Personality factors correlate with regional cerebral perfusion. Neuroimage 31, 489–495 10.1016/j.neuroimage.2005.12.048 16529951

[B59] Okada E. Delpy D. T. (2003a). Near-infrared light propagation in an adult head model. I. Modeling of low-level scattering in the cerebrospinal fluid layer. Appl. Opt. 42, 2906–2914 10.1364/AO.42.002906 12790439

[B60] Okada E. Delpy D. T. (2003b). Near-infrared light propagation in an adult head model. II. Effect of superficial tissue thickness on the sensitivity of the near-infrared spectroscopy signal. Appl. Opt. 42, 2915–2922 10.1364/AO.42.002915 12790440

[B61] Pan W. J. Thompson G. J. Magnuson M. E. Jaeger D. Keilholz S. (2013). Infraslow LFP correlates to resting-state fMRI BOLD signals. Neuroimage 74, 288–297 10.1016/j.neuroimage.2013.02.035 23481462 PMC3615090

[B62] Pan W. J. Thompson G. Magnuson M. Majeed W. Jaeger D. Keilholz S. (2011). Broadband local field potentials correlate with spontaneous fluctuations in functional magnetic resonance imaging signals in the rat somatosensory cortex under isoflurane anesthesia. Brain Connect. 1, 119–131 10.1089/brain.2011.0014 22433008 PMC3621847

[B63] Pierro M. L. Sassaroli A. Bergethon P. R. Ehrenberg B. L. Fantini S. (2012). Phase-amplitude investigation of spontaneous low-frequency oscillations of cerebral hemodynamics with near-infrared spectroscopy: a sleep study in human subjects. Neuroimage 63, 1571–1584 10.1016/j.neuroimage.2012.07.015 22820416 PMC3472105

[B64] Qin P. Grimm S. Duncan N. W. Holland G. Guo J. S. Fan Y. (2013). Self-specific stimuli interact differently than non-self-specific stimuli with eyes-open versus eyes-closed spontaneous activity in auditory cortex. Front. Hum. Neurosci. 7:437 10.3389/fnhum.2013.00437 23908625 PMC3725474

[B65] Quilty L. C. Godfrey K. M. Kennedy S. H. Bagby R. M. (2010). Harm avoidance as a mediator of treatment response to antidepressant treatment of patients with major depression. Psychother. Psychosom. 79, 116–122 10.1159/000276372 20090398

[B66] Raichle M. E. Macleod A. M. Snyder A. Z. Powers W. J. Gusnard D. A. Shulman G. L. (2001). A default mode of brain function. Proc. Natl. Acad. Sci. U.S.A. 98, 676–682 10.1073/pnas.98.2.676 11209064 PMC14647

[B67] Richter J. Brandstrom S. (2009). Personality disorder diagnosis using the temperament and character inventory. Compr. Psychiatry 50, 347–352 10.1016/j.comppsych.2008.09.002 19486733

[B68] Richter J. Eisemann M. Richter G. (2000). Temperament and character during the course of unipolar depression among inpatients. Eur. Arch. Psychiatry Clin. Neurosci. 250, 40–47 10.1007/PL00007538 10738864

[B69] Robinson O. J. Charney D. R. Overstreet C. Vytal K. Grillon C. (2012). The adaptive threat bias in anxiety: amygdala-dorsomedial prefrontal cortex coupling and aversive amplification. Neuroimage 60, 523–529 10.1016/j.neuroimage.2011.11.096 22178453 PMC3288162

[B70] Rosellini A. J. Brown T. A. (2011). The NEO Five-Factor Inventory: latent structure and relationships with dimensions of anxiety and depressive disorders in a large clinical sample. Assessment 18, 27–38 10.1177/1073191110382848 20881102 PMC5639474

[B71] Sassaroli A. Pierro M. Bergethon P. R. Fantini S. (2012). Low-frequency spontaneous oscillations of cerebral hemodynamics investigated with near-infrared spectroscopy: a review. IEEE J. Sel. Top. Quantum Electron. 18, 1478–1492 10.1109/JSTQE.2012.2183581 11112395

[B72] Scholvinck M. L. Maier A. Ye F. Q. Duyn J. H. Leopold D. A. (2010). Neural basis of global resting-state fMRI activity. Proc. Natl. Acad. Sci. U.S.A. 107, 10238–10243 10.1073/pnas.0913110107 20439733 PMC2890438

[B73] Schroeter M. L. Schmiedel O. Von Cramon D. Y. (2004). Spontaneous low-frequency oscillations decline in the aging brain. J. Cereb. Blood Flow Metab. 24, 1183–1191 10.1097/01.WCB.0000135231.90164.40 15529019

[B74] Sher K. J. Bartholow B. D. Wood M. D. (2000). Personality and substance use disorders: a prospective study. J. Consult. Clin. Psychol. 68, 818–829 10.1037/0022-006X.68.5.818 11068968

[B75] Singh A. K. Okamoto M. Dan H. Jurcak V. Dan I. (2005). Spatial registration of multichannel multi-subject fNIRS data to MNI space without MRI. Neuroimage 27, 842–851 10.1016/j.neuroimage.2005.05.019 15979346

[B76] Smith D. J. Duffy L. Stewart M. E. Muir W. J. Blackwood D. H. (2005). High harm avoidance and low self-directedness in euthymic young adults with recurrent, early onset depression. J. Affect. Disord. 87, 83–89 10.1016/j.jad.2005.03.014 15967233

[B77] Sugiura M. Kawashima R. Nakagawa M. Okada K. Sato T. Goto R. (2000). Correlation between human personality and neural activity in cerebral cortex. Neuroimage 11, 541–546 10.1006/nimg.2000.0564 10806039

[B78] Tachtsidis I. Elwell C. E. Leung T. S. Lee C.-W. Smith M. Delpy D. T. (2004). Investigation of cerebral haemodynamics by near-infrared spectroscopy in young healthy volunteers reveals posture-dependent spontaneous oscillations. Physiol. Meas. 25, 437–445 10.1088/0967-3334/25/2/003 15132309

[B79] Tan B. Kong X. Yang P. Jin Z. Li L. (2013). The difference of brain functional connectivity between eyes-closed and eyes-open using graph theoretical analysis. Comput. Math. Methods Med. 2013, 976365 10.1155/2013/976365 23690886 PMC3652100

[B80] Toronov V. Webb A. Choi J. H. Wolf M. Michalos A. Gratton E. (2001). Investigation of human brain hemodynamics by simultaneous near-infrared spectroscopy and functional magnetic resonance imaging. Med. Phys. 28, 521–527 10.1118/1.1354627 11339749

[B81] Valencia M. Artieda J. Bolam J. P. Mena-Segovia J. (2013). Dynamic interaction of spindles and gamma activity during cortical slow oscillations and its modulation by subcortical afferents. PLoS ONE 8:e67540 10.1371/journal.pone.0067540 23844020 PMC3699652

[B82] Vidal-Gonzalez I. Vidal-Gonzalez B. Rauch S. L. Quirk G. J. (2006). Microstimulation reveals opposing influences of prelimbic and infralimbic cortex on the expression of conditioned fear. Learn. Mem. 13, 728–733 10.1101/lm.306106 17142302 PMC1783626

[B83] Wang J. Qin W. Liu B. Zhou Y. Wang D. Zhang Y. (2013). Neural mechanisms of oxytocin receptor gene mediating anxiety-related temperament. Brain Struct. Funct. [Epub ahead of print]. 10.1007/s00429-013-0584-9 23708061

[B84] Wang L. Dai W. Su Y. Wang G. Tan Y. Jin Z. (2012a). Amplitude of low-frequency oscillations in first-episode, treatment-naive patients with major depressive disorder: a resting-state functional MRI Study. PLoS ONE 7:e48658 10.1371/journal.pone.0048658 23119084 PMC3485382

[B85] Wang L. Saalmann Y. B. Pinsk M. A. Arcaro M. J. Kastner S. (2012b). Electrophysiological low-frequency coherence and cross-frequency coupling contribute to BOLD connectivity. Neuron 76, 1010–1020 10.1016/j.neuron.2012.09.033 23217748 PMC3531830

[B86] Wei L. Duan X. Zheng C. Wang S. Gao Q. Zhang Z. (2012). [Epub ahead of print]. Specific frequency bands of amplitude low-frequency oscillation encodes personality. Hum. Brain Mapp. 10.1002/hbm.22176 PMC6869309 22987723

[B87] Welch P. (1967). The use of fast Fourier transform for the estimation of power spectra: a method based on time averaging over short, modified periodograms. IEEE Trans. Audio Electroacoustics 15, 70–73 10.1109/TAU.1967.1161901

[B88] Won Kim S. Grant J. E. (2001). Personality dimensions in pathological gambling disorder and obsessive–compulsive disorder. Psychiatry Res. 104, 205–212 10.1016/S0165-1781(01)00327-4 11728609

[B89] Yan C. Liu D. He Y. Zou Q. Zhu C. Zuo X. (2009). Spontaneous brain activity in the default mode network is sensitive to different resting-state conditions with limited cognitive load. PLoS ONE 4:e5743 10.1371/journal.pone.0005743 19492040 PMC2683943

[B90] Yang H. Long X. Y. Yang Y. Yan H. Zhu C. Z. Zhou X. P. (2007). Amplitude of low frequency fluctuation within visual areas revealed by resting-state functional MRI. Neuroimage 36, 144–152 10.1016/j.neuroimage.2007.01.054 17434757

[B91] Yin Y. Li L. Jin C. Hu X. Duan L. Eyler L. T. (2011). Abnormal baseline brain activity in posttraumatic stress disorder: a resting-state functional magnetic resonance imaging study. Neurosci. Lett. 498, 185–189 10.1016/j.neulet.2011.02.069 21376785

[B92] Youn T. Lyoo I. K. Kim J. K. Park H. J. Ha K. S. Lee D. S. (2002). Relationship between personality trait and regional cerebral glucose metabolism assessed with positron emission tomography. Biol. Psychol. 60, 109–120 10.1016/S0301-0511(02)00047-9 12270587

[B93] Zou Q. H. Zhu C. Z. Yang Y. Zuo X. N. Long X. Y. Cao Q. J. (2008). An improved approach to detection of amplitude of low-frequency fluctuation (ALFF) for resting-state fMRI: fractional ALFF. J. Neurosci. Methods 172, 137–141 10.1016/j.jneumeth.2008.04.012 18501969 PMC3902859

[B94] Zuckerman M. Cloninger C. R. (1996). Relationships between Cloninger's, Zuckerman's, and Eysenck's dimensions of personality. Pers. Individ. Dif. 21, 283–285 10.1016/0191-8869(96)00042-6 PMC4486314 26146428

